# Non-adherence to oral antibiotics for community paediatric pneumonia treatment in Malawi – A qualitative investigation

**DOI:** 10.1371/journal.pone.0206404

**Published:** 2018-10-31

**Authors:** Carina King, Rebecca Nightingale, Tambosi Phiri, Beatiwel Zadutsa, Esther Kainja, Charles Makwenda, Tim Colbourn, Fiona Stevenson

**Affiliations:** 1 Institute for Global Health, University College London, London, United Kingdom; 2 Liverpool School of Tropical Medicine, Liverpool, United Kingdom; 3 Parent and Child Health Initiative, Lilongwe, Malawi; 4 Research Department of Primary Care and Population Health, University College London, London, United Kingdom; University of South Florida, UNITED STATES

## Abstract

**Background:**

Pneumonia remains the leading cause of paediatric infectious mortality globally. Treatment failure, which can result from non-adherence to oral antibiotics, can lead to poor outcomes and therefore improving adherence could be a strategy to reduce pneumonia related morbidity and mortality. However, there is little published evidence from low-resource settings for the drivers of non-adherence to oral antibiotics in children.

**Objective:**

We aimed to investigate reasons for adherence and non-adherence in children diagnosed and treated in the community with fast-breathing pneumonia in rural Malawi.

**Methods:**

We conducted focus group discussions (FGDs) with caregivers of children known to have been diagnosed and treated with oral antibiotics for fast-breathing pneumonia in the community and key informant interviews with community healthcare workers (CHW). FGDs and interviews were conducted within communities in Chichewa, the local language. We used a framework approach to analyze the transcripts.

**Results:**

We conducted 4 FGDs with caregivers and 10 interviews with CHWs. We identified four themes, which were common across caregivers and CHWs: knowledge and understanding, effort, medication perceptions and community influences. Caregivers and CHWs demonstrated good knowledge of pneumonia and types of treatment, but caregivers showed confusion around dosing and treatment durations. Effort was needed to seek care, prepare medication and understand regimens, acting as a barrier to adherence. Perceptions of how well the treatment was working influenced adherence, with both quick recovery and slow recovery leading to non-adherence. Community influences were both supportive, with transport assistance for referrals and home visits to improve adherence, and detrimental, with pressure to share treatments.

**Conclusion:**

Adherence to oral antibiotic treatment for fast-breathing pneumonia was understood to be important, however considerable barriers we described within this rural low-resource setting, such as the effort preparing and administering medication, community pressures to share drugs and potential complexity of regimens.

## Introduction

Despite significant progress in reducing pneumonia-related morbidity and mortality, it remains the leading cause of infectious deaths among children under-five globally [1, 2]. Several factors contribute to the high pneumonia burden, including: poor access to treatment; poor care-seeking behaviours; and treatment failure [3]. Treatment failure—the persistence or progression to severe illness following the completion of treatment, is associated with worse outcomes in children with pneumonia [4]. A systematic review of paediatric treatment failure in fast-breathing pneumonia from developing countries found rates ranging from 7.8–22.9% [5]. A study from Malawi reported a treatment failure rate of 15% amongst pediatric fast-breathing pneumonia cases [6]. Therefore, this poses a considerable opportunity for improved case management and treatment.

There are multiple reported reasons for treatment failure from low-resource settings, including a lack of differentiation between viral and bacterial causes of pneumonia, and non-adherence to antibiotic treatment [7, 8]. Non-adherence is the late or non-initiation of treatment, sub optimal implementation of the dosing regimen or early discontinuation [9]. Definitions of adherence in this context vary, but commonly include either 80% or 100% dose completion within the correct timeframe. There is a considerable gap in published research into drivers and barriers to adherence to short-term antibiotics in children from low-income settings, especially social and cultural factors. Available evidence reports a wide range of adherence rates, from 3–60% [10–13]; however these varied in terms of the antibiotics studied and context of their use, making it challenging to generalize to paediatric pneumonia. Recent data from Malawi found 20% of children completed less than 100% of the oral antibiotic treatment course, and one in ten took less than 80% of their treatment for fast-breathing pneumonia in the community [14]. Reported reasons for non-adherence in respiratory infections include: duration and number of doses, unfriendly formulations (e.g. large tables vs. syrups), healthcare provider relationship, and illness severity [15, 16]. While there has been research into adherence for longer-term non-antibiotic treatments for chronic diseases (e.g. HIV) and paediatric malaria [17, 18], data relating specifically to short-term antibiotics in low-resource settings remains limited.

Quantitative analysis from Malawi found that being given multiple drugs (either for pneumonia or an additional concurrent diagnosis) and being sicker for longer before treatment initiation were associated with non-adherence to oral antibiotics in children under-5 years in the community [14]. However, this gives us a limited understanding of what is driving caregiver behaviour in relation to treatment adherence, and what approaches could be used to improve adherence. Improving adherence to oral antibiotics could provide an opportunity for improving paediatric outcomes, especially for outpatient treatments managed by caregivers in rural community settings [7, 8]. In order to develop context appropriate and effective strategies to improve adherence rates for home-based care, we need a better understanding of how these treatments are managed, and motivations and barriers to completing prescribed antibiotics in paediatric pneumonia. We aimed to gain an understanding of both caregiver and healthcare provider experiences and perspectives of the drivers of adherence to oral antibiotics in children under-5 years in a rural, low-resource setting.

Malawi, despite having successfully reduced under-5 mortality by 2/3rds from 1990 to achieve Millennium Development Goal 4 [19], continues to have a high under-5 mortality of 64/1,000 livebirths [20]. This study was conducted in Mchinji district, central region of Malawi, with a population of 500,000, of which 80% live in rural areas with a subsistence farming economy. Primary care is free and provided by government employed community healthcare workers (CHW), known locally as Health Surveillance Assistants. CHWs receive 10-weeks training, which includes integrated community case management (iCCM) for common pediatric infections, including pneumonia, malaria, diarrhea and conjunctivitis [21, 22] (Table 1). In-line with iCCM guidelines fast-breathing pneumonia is treated in the community, and pneumonia with chest indrawing or general danger signs referred to health centres. The primary treatment regime for fast-breathing pneumonia changed from 5-days of cotrimoxazole to 5-days of amoxicillin in 2015, in accordance with WHO recommendations [23]. CHWs are generally recruited from and live within the communities they serve and conduct weekly or bi-weekly village clinics, and home visits.

**Table 1:** Integrated community case management treatment guidelines for Malawi for children aged 2–59 months old [23].

Diagnosis	Medication	Duration	Frequency	Dose
Pneumonia	Amoxicillin	5 days	Twice daily	2–11 months: 1 tablet 12–59 months: 2 tablets
	Cotrimoxazole (‘bactrim’)	5 days	Twice daily	2–11 months: ½ tablet 12–59 months: 1 tablet
	Paracetamol	3 days	Three times daily	<10kg: ¼ tablet >10kg: ½ tablet
Malaria	Lumefantrine-artemether (LA)	3 days	Twice daily	2–35 months: 1 tablet 36–59 months: 2 tablets
	Paracetamol	3 days	Three times daily	<10kg: ¼ tablet >10kg: ½ tablet
Diarrhoea	Oral rehydration salts (ORS)	Unspecified	After each loose stool	At least ½ cup
	Zinc supplement	10 days	Once daily	2–6 months: ½ tablet 7–59 months: 1 tablet

## Methods

We conducted semi-structured interviews with healthcare providers and focus group discussions (FGDs) with caregivers in Mchinji district, Malawi, in February 2017. FGDs were chosen to explore commonalities and shared experiences between caregivers, while semi-structured interviews with CHWs were chosen to allow for individual understanding and experiences to be shared on diagnosing and treating paediatric infections, and to encourage CHWs to feel more comfortable in discussing challenges.

### Study context

In 2013–2014 we conducted a prospective cohort study of children aged 2–59 months diagnosed with fast-breathing pneumonia (‘pneumonia’), treated in the community [6]. Children were followed-up in their homes 5-days later and asked about concurrent diagnoses and treatments, additional care-seeking, and completion of antibiotics. In this population, 51% of caregivers reported their child had been diagnosed with a concurrent infection to pneumonia which they received treatment for [14]. Adherence, which was self-reported, was verified using pill counts in 30% of interviews.

### Recruitment

We purposively recruited both caregivers and CHWs, based on reported adherence from the prospective cohort study described above [6]. We selected the two village clinics with the highest proportion of non-adherent cases and the two with the lowest proportion of non-adherent cases. We listed households known from the previous cohort study which had a child who had been diagnosed with and treated in the community for pneumonia. The mothers (or primary caregiver if the mother was not available) were selected to participate in the FGDs. We targeted 7–8 caregivers from each village, for each of four FGDs. Participants were invited in person, through engagement with village leaders and CHWs.

We selected five CHWs who were included in the cohort study and had received additional training and mentorship on paediatric pneumonia. We selected five CHWs from non-study areas who had not received this additional support to elicit a wider range of experiences. Those CHWs from the study area were selected to represent those with the highest (‘adherent’) and lowest (‘non-adherent’) proportions of adherent cases in their catchment areas, including those CHW from villages where we recruited caregivers. The five ‘non-study’ CHW were selected in collaboration with the local health management team to represent a range of clinic sizes, locations and distance from health centres. All CHWs were invited by phone by study staff.

### Data collection

Interviews and FGDs were led by a local female researcher (EK) with experience of qualitative research, and supported by a local male clinical researcher (BZ) with knowledge of pneumonia and iCCM guidelines. The interviews and FGDs were conducted within the communities, and followed topic guides including: understanding of pneumonia and concurrent infections, understanding of antibiotics and concurrent treatments, and motivations and barriers to adherence (S1 Supporting Information). All interviews and FGDs were conducted in Chichewa, the local language, and were audio recorded following consent. Recordings and notes were transcribed, translated and the final transcripts discussed and agreed upon by BZ and EK. Participants were reimbursed for their travel costs and provided with refreshments, but not offered any other financial incentives.

### Analysis

We used a framework analysis approach based on the five steps described by Gale et al. (2013): familiarization, coding, developing and applying a framework, charting and interpretation [24]. The transcripts were initially printed and read through by two researchers (CK and RN) for familiarization. Following familiarization, CK and RN discussed and agreed on major codes for both the FGDs and interviews, based on the structure of the topic guides (e.g. “understanding of antibiotics”). The data were then coded independently in Microsoft Excel. These codes were shared and a thematic framework was developed through a round table discussion until a consensus was reached on the mapping of the codes by FS, TC, RN and CK. The themes and their interpretation were shared and discussed with the two Malawian researchers who collected the data (BZ and EK) to ensure it was culturally appropriate and incorporated their perceptions.

### Ethics

We sought informed written consent, following an explanation of the study aim, for all the FGDs and interviews. This study was approved by the Malawi National Health Sciences Research Ethics Committee [protocol number: 16/4/1569] and the University College London research ethics committee [reference: 8075/002].

## Results

Four FGDs with a combined total of 24 caregiver participants (Table 2) and 10 interviews with CHWs were conducted (non-adherent = 4; adherent = 1; non-study = 5). We identified four themes: knowledge and understanding, effort, medication perceptions and community influences. These themes were common to caregiver and healthcare provider discussions; therefore, we have presented these perspectives together.

**Table 2:** Summary of caregivers included in the focus group discussions.

Group	Number (N = 24)	Education (n, %)	Age (mean, range)
Adherent	12	None = 1 (8%) Primary = 8 (67%) Secondary = 3 (25%)	33 (23–45)
Non-adherent	12	Primary = 10 (83%) Secondary = 2 (17%)	31 (21–43)

### Knowledge and understanding

Both caregiver and CHW knowledge about pneumonia and its treatments, and how they understood these, were significant for adherence. Caregivers had good knowledge of pneumonia and the importance of treatment, and all participants demonstrated understanding of the potential consequences of non-adherence, including: drug resistance, recurrent infections and onward transmission. This appreciation of the increased risk of poorer outcomes and the desire for children to recover were key drivers of adherence.

*"It is a dangerous disease*, *especially if you have not followed instructions on giving treatment*, *a child can pass on [die]*, *but if you properly follow the instructions on how to give the drugs*, *pneumonia in the child can be assisted”* (Caregiver 1, non-adherent)

Knowledge of specific treatments and their regimes was mixed. In all groups, caregivers could name an antibiotic given for pneumonia, as well as other common drugs for malaria or diarrhoea. With differing regimes and multiple treatments for different illnesses, having concurrent infections can result in complex treatments. We have previously found that concurrent infections are common in this population (51%), but CHWs had mixed perceptions, stating they occurred ‘rarely’ to being ‘very common’. Confusion regarding dosing and duration of treatments was apparent among caregivers, and even a CHW incorrectly stated pneumonia treatment was 3-days.

Communication between CHWs and caregivers was a key factor for adherence, with a lack of effective communication resulting in caregiver confusion around treatment regimes. CHWs described a range of education strategies they use for improving adherence, including: demonstrations, asking caregivers to repeat back the recommended treatment, and providing strategies for illiterate caregivers or those without access to a clock (e.g. turning on a radio). However, CHWs acknowledged that caregivers could forget, were unable to understand due to poor education, or become confused; one caregiver attributed this confusion to CHWs:

"*That’s the way the [CHW] tells us*, *so we follow*. *If we are wrong it is the [CHW] who makes us go wrong*" (Caregiver 4, non-adherent)

CHWs use a standardized case report tool, called the ‘sick child reporting form’, and this was highlighted as an integral aid in diagnosis, treatment and referral decision-making. They demonstrated good knowledge of danger signs for referral and clinical features of common illnesses, utilizing this ‘checklist’ approach.

"*We do not just write the drugs with no basis*, *we follow what is shown on the sick child recording form where we tick the history taken and find the problem*, *after that we make a decision*" (CHW 5, non-adherent)

There was a lack of clarity amongst CHWs regarding a change to the national treatment guidelines for paediatric pneumonia, and a change in the antibiotic of choice from cotrimoxazole to amoxicillin. CHWs reported currently using different antibiotics due to stocking issues, but crucially the reason for the change in treatment was poorly understood. The main reason given was that the national-level source of funding for drug procurement had changed, and therefore the antibiotic had changed.

"*We only give this drug called cotrimoxazole*, *although they introduced amoxicillin which was made available once*, *but up to now they are no longer provided*" (CHW 3, non-adherent)

### Effort

The theme of ‘effort’ was common for many of the processes around treatment, relating to the work that is involved in administering and preparing medications, the effort needed to understand complex regimes and the time required to seek and attend care. The term that was most commonly used by caregivers and CHWs to capture this barrier to adherence was “laziness”:

"*…the decision is based on laziness*, *on giving the child drugs*, *which can lead to the child not getting well"* (Caregiver 4, non-adherent)

In this context however, the term ‘laziness’ is more reflective of the ability to cope with daily competing priorities, rather than an unwillingness to spend time and effort on multiple activities. This was highlighted by caregivers describing the time and resource involved in giving the treatments, such as preparing food to eat with the medication, the challenge of getting a child to take the medication, and balancing this time with the need to conduct their routine daily tasks.

"*Sometimes it is the child that is a problem on giving the treatment*" (Caregiver **2**, non-adherent)

"A*t times we are busy*, *which is what makes you forget to give the child drugs*" (Caregiver 10, adherent)

“*When Bactrim has been given you are told in the morning to cook porridge and give it*. *If [the child] is small*, *we cut [the tablet] and give it*. *In the afternoon after you have given food*, *cut [the tablet] and give it to the child*, *in the evening give them food and cut [the tablet] and give it*.” (Caregiver **5**, non-adherent)

When given multiple medications, which require different preparations (e.g. dividing tablets or diluting oral rehydration salts (ORS) in water), caregivers described the considerable effort it takes to ensure correct preparation and timing, considering the additional resources that may also be needed for this (e.g. food).

The effort involved in seeking care was a barrier to attending, but conversely did not prevent caregivers attending referrals when the seriousness of the condition was understood. However, there was some disparity between CHW and caregiver accounts, highlighting the difference between understanding importance and prioritizing action.

*"When the health worker tells me to go to the district hospital*, *I get worried*, *then we see the condition of the child has warranted referral*, *thus why we need [to attend the] referral to get treatment quickly"* (Caregiver 13, adherent)

*"When we look at people in Mchinji*, *like this time they are very busy in the garden [farming] tobacco*, *that a child is very sick and they should take it to the hospital*, *they feel they will lose time going to the hospital with the child"* (CHW 9, adherent)

### Medication perceptions

The ways in which treatment was perceived, both positively, negatively and in relation to effectiveness, influenced adherence. We found an interesting contradiction as both non-recovery and quick recovery were given as reasons for non-adherence. With non-recovery, caregivers indicated that if no improvement was observed then treatment would not necessarily be continued.

"*What happens to make you stop giving drugs is that you are giving the drugs and you find that the child is not improving*, *so they say no that’s all I will just stop giving this drug*" (Caregiver 8, adherent)

Conversely, caregivers (presented as the actions of ‘the other’) and CHWs discussed the quick recovery of children leading to the cessation of treatment, relating to ‘laziness’ as the effort of giving treatment that’s perceived as not needed or not working was burdensome.

"*After starting treatment*, *the child is able to play around*, *they have the confidence that the child has improved when in reality the child is still sick*" (CHW 5, non-adherent)

In Malawi there is a pluralist system of medicine, where traditional medicine operates alongside public and private provision of Western medicine. Given the choice of Western medicine or traditional medicine, one caregiver stated that some mothers (again described as the actions of ‘the other’) would opt for the later. Although interestingly care would still be sought from both, highlighting the everyday operation of medical pluralism in this setting. This was only raised by one group of caregivers, and was not acknowledged by CHWs.

"*When the child is sick with pneumonia they go to the witch doctor*, *there they are told your child was thrown to the ground by witches and when they visit the hospital they are told the child has malaria*. *They prefer the witch doctor’s advice and leave the drugs*" (Caregiver 22, adherent)

One group discussed a preference for injections, which are not part of the Malawi treatment guidelines [23], but are available from private facilities. This implied dissatisfaction with the standard treatment given freely by government facilities, and the potential for the pluralist nature of health service provision to influence adherence.

*"When you go to these government hospitals no matter how sick the child they just give you panadol and bactrim*. *When you go to paying hospitals it’s where you get injections" (*Caregiver 9, adherent)

### Community influences

Community influences were discussed around pressures to share medications, and community solutions to adherence and referrals. Village clinics only provide care for children under-5 years as the group at highest risk of severe disease and poor outcomes; older children, such as siblings or neighbours’ children, would need to seek care at higher-level facilities which requires travel and resources. Both caregivers and CHWs discussed the behaviour of sharing medications or keeping spares for themselves or their neighbours. Caregivers stated that sharing was not approved of, as they understood the dangers of giving drugs without a clinical assessment, but it was accepted that this was a common practice.

"*The one who shares the drugs thinks she is merciful and is helpful to the friend*, *while the receiving person just receives [the drugs] without knowing that the condition could be different*" (Caregiver 15, non-adherent)

This suggests a ‘gift relationship’ and that there is also an element of expectation and reciprocity. CHWs mirrored this concern and several of them discussed strategies that they were implementing to try and understand this issue and address it, such as home-based follow-ups.

"*…because of the follow-ups*, *we found that mothers most of the time when they take the drugs they share them with others who maybe are also sick in the village*" (CHW 10, non-adherent)

Many CHWs described having the support of village health committees who work with them to conduct follow-ups of children being treated in the community at day 3. Some CHWs reportedly only gave 3-days of antibiotics instead of 5, with the remainder given during these household visits. Village health committees were reported as providing a supportive role in helping caregiver’s take their children for referrals, for example loaning them a community-owned bicycle for transportation. However, this was not uniform across all communities as some CHWs stated that transport was the responsibility of the caregiver alone. These highlight a lack of consistency in supportive care and between practice and health education messaging, between CHWs.

"*If the guardian has complained that he doesn’t have transport that becomes our work together with the committee to help them find transport so that the child should receive that treatment*" (CHW 6, non-study)

## Discussion

We identified several drivers for non-adherence to short-term oral antibiotic treatment for paediatric pneumonia, in a rural low-resource setting in sub-Saharan Africa. These included the effort needed to both understand and then administer sometimes complex treatment regimes, the perception that the treatment wasn’t working, or that the child had recovered, and social pressures to share medications. On the other hand, drivers for adherence related to the desire for children to recover and the appreciation of the severity of childhood infections. Several local solutions, such as caregiver education, household follow-ups and engagement with village health committees, were presented to try and improve adherence, suggesting it is considered an important issue by CHWs.

Many of these themes are similar to those reported by previous studies looking at both long-term and short-term antibiotics and malaria treatments in children. Frequently cited in the literature is improved adherence with ‘patient friendly’ drugs [16]. A study from Ghana in children with malaria found 91% adherence to pre-packaged chloroquine tablets versus 42% for syrup, which required each dose to be measured out and then prepared [18]. We found this similar barrier of ‘effort’, with caregivers describing the need to prepare medications (e.g. splitting tablets: “*According to age*, *children are given half*, *quarter or full tablet*” Caregiver 23, Adherent). Additionally, in high-resource settings, the taste, texture and size of oral medications are shown to affect adherence [13, 25, 26]. Considering the wider evidence that medication formulations affect adherence, reformulating antibiotics to be age appropriate could reduce this treatment burden, such as different pre-packaged pill sizes for different ages. While this was only one element of the work needed to correctly give treatment, strategies to streamline treatments may prove effective in reducing cognitive and time burdens for caregivers.

We did not find considerable differences between barriers posed by caregivers and those given by CHWs to treatment completion, nor between those with high reported rates of adherence and non-adherence. However, we did see examples of disconnect between knowledge and practice, and much of this related to effort and social pressures. While caregivers acknowledged that completing treatment was important, the cultural norm of sharing and gifting with neighbours exerts both strong motivations of reciprocity and the potential for social sanctions if not followed [27]. This has been observed in other settings, with drug sharing reported for HIV treatment in rural Uganda, with participants reporting that they anticipated being pressured into sharing their drugs [28]. However, this has not been reported previously for short-term antibiotic treatments from similar settings.

Interestingly, while CHWs recognized the potential for confusion in the effort needed to understand concurrent diagnoses and therefore treatment regimens, caregivers did not raise this. We have previously found that children with a concurrent diagnosis had 75% (95% CI: 1.00–3.07) higher odds of being non-adherent [14]. While this link between having multiple diagnoses and treatments and failing to complete all treatments was not discussed directly, it was apparent from caregiver discussions that the differing treatment durations for pneumonia (5 days of amoxicillin/cotrimoxazole) and malaria (3 days of Lumefantrine Artemether (LA)–summarized in Table 1) was an area of confusion. There is evidence from randomized controlled trials that there is no difference in treatment failure rates for fast-breathing pneumonia when comparing 3 versus 5-days of amoxicillin [8, 29]. Further research into the possible impact of changing the 5-day treatment course to 3-days on adherence rates, especially in children with concurrent malaria infections would be valuable. Additionally, supportive CHW training focused on more complex community management of concurrent infections, beyond a ‘checklist’ approach, could be incorporated into the iCCM programme.

The main limitation in this study was the potential for social desirability bias, where participants provide responses which they think the facilitators want to hear. In two of the FGDs, caregivers initially insisted that the idea of not completing treatment would not occur to them and offered no reasons for non-adherence. However, when framed as their ‘friends or neighbours’ they offered several hypothetical reasons. This suggests that they were uncomfortable acknowledging a negatively perceived personal behaviour amongst their peers or the researchers, but when attributable to ‘the other’ this was not an issue. While this resulted in fewer personal experiences shared, participants offered both motivations and barriers to adherence, and similar reasoning was given across groups and with CHWs. This may have also influenced the various strategies which CHWs stated they implemented for tackling non-adherence, such as conducting the recommended follow-up of children on day 3. While there may be a disconnect between what they state in practice and what occurs in reality, the discussions suggested that non-adherence is known about and acknowledged as an issue. Additionally, the number of FGDs and interviews conducted was determined *a priori*, rather than taking a saturation approach. Conducting additional interviews or FGDs may have resulted in new themes or additional interpretations.

These data suggest there may be several pragmatic approaches to improving adherence within this setting, such as simplifying the treatment regimens for multiple concurrent infections (e.g. 3-day antibiotic regimens), or reducing the amount of effort required in preparing the doses with age-appropriate pill formulations and food supplements. However, the effort required in managing acute paediatric infections in the community by caregivers was multifaceted and considerable. Therefore, a complex solution will likely be needed to this complex issue. While several local solutions have been described, and knowledge was generally high amongst caregivers, there was still a gap between knowledge, understanding and behaviour. A key finding was the existence of a gifting relationship, exerting social pressure to not complete short term antibiotic courses. This suggests that an intervention combining both guideline and medication reformulation, alongside community engagement and mobilization approaches to change the culture of sharing medications would be needed to address adherence within a rural community setting.

## Supporting information

S1 Supporting Information Topic guides. (DOCX)

S2 Supporting Information COREQ checklist. (DOCX)
