# Supplementary material for: Non-adherence to oral antibiotics for community paediatric pneumonia treatment in Malawi – A qualitative investigation
Source: PLoS One. 2018 Oct 31;13(10):e0206404. doi: 10.1371/journal.pone.0206404 (PMC6209296; doi:10.1371/journal.pone.0206404)
Supplement: S1 Supporting Information — (DOCX) [file pone.0206404.s001.docx]

*Topic Guides:*

**Focus Group Discussions with care-givers of children diagnosed with non-severe pneumonia**

- - What is your understanding of pneumonia?
  - Has your child ever been diagnosed with pneumonia? Can you describe what happened, or what you did?
  - Is pneumonia a serious illness?
  - What are antibiotics? [Probe: give example of Bactrim]
  - What is your understanding about how antibiotics should be used?
- What about other drugs, is it any different? [Probe: give example of LA and ORS]
- Where do you get the information about how they should be given?
  - Do you think it is important to finish all the tablets given? Why/why not?
- Is this any different if you have multiple medications? Why/why not?
  - What factors influence your decision to give (and not give) your child the treatment?
  - What factors would make you less likely to give your child the treatment?
  - What factors would influence your decision to go to the hospital or health centre if the CHW referred you?
- What is the most important factor in the decision to go for a referral or not?

**Semi-structured Interviews with Community Healthcare workers:**

- What is your understanding of antibiotics? Can you give an example of when you would prescribe antibiotics for a child?
- What is your understanding about how antibiotics should be used?
- What is your understanding of multiple infections in children?
- How often do you see this in children in your clinic?
- What factors would encourage you to prescribe more than one medication?
- If you prescribe more than one medication do you think it affects how the treatment is used?
- Explain how you communicate information about treatment?
- [Probe for information about taking all of the doses]
- How do you decide whether to refer someone, or give them treatment?
- [Probe: not just clinical symptoms, but other factors such as e.g. transport].
- Are there any factors that would stop you from giving treatment, or making a referral?
